# Genetic parameters and litter trait trends of Danish pigs in South Vietnam

**DOI:** 10.5713/ab.20.0692

**Published:** 2021-06-23

**Authors:** Nguyen Huu Tinh, Tran Van Hao, Anh Phu Nam Bui

**Affiliations:** 1 Department of Animal Genetics, Institute of Animal Sciences for Southern Vietnam, Binh Duong 75000, Vietnam; 2 Faculty of Biotechnology, Ho Chi Minh City Open University, Ho Chi Minh City 74000, Vietnam

**Keywords:** Correlation, Danish Pig, Genetic Trend, Heritability, Litter Trait, Variance

## Abstract

**Objective:**

The objective of this study was to estimate the genetic parameters and various litter trait trends of Danish pigs in South Vietnam, including the number born alive (NBA), number weaned (NW), and litter weight at the 21st day (LW21).

**Methods:**

Records of 936 Yorkshire sows with 3361 litters and 973 Landrace sows with 3161 litters were used to estimate the variance components, genetic parameters, and trends of NBA, NW, and LW21. The restricted maximum likelihood method was applied using VCE6 software to obtain the variance components and genetic parameters. Thereafter, the best linear unbiased prediction procedure with an animal model was applied using PEST software to estimate the breeding values of the studied traits.

**Results:**

The heritability estimates were low, ranging from 0.12 to 0.21 for NBA, 0.03 to 0.04 for NW, and from 0.11 to 0.13 for LW21. The genetic correlation between the NBA and NW was relatively strong in both breeds, at 0.77 and 0.60 for Yorkshire and Landrace, respectively. Similarly, the genetic correlation between the NW and LW21 was considerably stronger in Landrace pigs (0.71) than in Yorkshire pigs (0.48). The estimates of annual genetic progress were 0.0431, 0.0233, and 0.0461 for NBA, NW, and LW21 in Landrace pigs and 0434, 0.0202, and 0.0667 for NBA, NW, and LW21 in Yorkshire pigs, respectively.

**Conclusion:**

The positive genetic trends estimated for the additive genetic values of the selected traits indicated that the current breeding system has achieved favorable results.

## INTRODUCTION

Genetic parameters are influenced by the genetic structure of animal populations and may, therefore, differ from one population to another [1]. In pigs, the heritability estimates of production and reproduction traits vary with population and time. The heritability of litter traits in most herds is between 0.10 and 0.20 [2], but it can be less than 0.10 [3]. Hence, precise estimates of variance components and selective trait heritability are essential to ensure an efficient breeding program for certain populations.

For organized breeding programs, estimates of the genetic trends of selection traits achieved after several generations through selection schemes are crucial to describe the genetic progress, assess selection effectiveness, and adjust the breeding objectives if necessary. In most cases, the annual means of the predicted breeding values of selected lines can be used to estimate the genetic trends of objective traits in selection programs [4].

In recent decades, the genetics of Yorkshire, Landrace, Duroc and Pietrain pigs have been imported into Vietnam from the USA, Canada, Australia, and other countries [5]. The variance components, and production and reproduction trait heritability have been estimated in genetic evaluation programs for pigs [6,7]. Since 2013, the genetics of Danish Yorkshire and Landrace pigs have been introduced to Vietnam under the Official Development Assistant (ODA) project of the Denmark Government. Danish Yorkshire and Landrace pigs are known to exhibit a very high potential for reproduction; therefore, these pigs have since been adapted and selected for genetic improvements in litter traits under the tropical conditions of South Vietnam [8]. After several selective generations, in this study, we attempted to estimate the heritability and describe the genetic progress of litter traits in Danish Yorkshire and Landrace pigs. Estimating the genetic parameters and assessing the genetic trends of these pigs are key for developing an efficient breeding program in the future.

## MATERIALS AND METHODS

### Ethics statement

Samples collected were used only for routine diagnostic purpose of the breeding programs and not specifically for the purpose of this project. Therefore, approval of an ethics committee was not mandatory. Sample collection and data recording were conducted strictly according to the Vietnamese law on animal protection and welfare.

### Animals and data collection

The research was conducted on purebred Danish Yorkshire and Landrace pigs (DanAvl Yorkshire and DanAvl Landrace) imported from Denmark in 2013, with an initial number of 233 animals, including 122 Yorkshire pigs (19 male and 103 female) and 156 Landrace pigs (30 male and 126 female). Since then, the pig genetics have been nurtured and purebred in the Binh Minh National Pig Breeding Farm in South Vietnam. Gilts, boars, and pregnant sows were maintained in individual stalls in a closed-house system, with an ambient temperature of 28°C to 30°C and relative humidity of 60% to 70%. Farrowing sows were maintained in both closed-house and open-house systems. The open-house system was naturally ventilated with a day and night temperature amplitude of 24°C to 34°C and a relative humidity ranging from 50% to 60% during the dry season (November to May) and 80% to 90% during the wet season (June to October). Gilts and non-lactating sows were fed 2.4 to 2.6 kg of feed containing 16% crude protein and total calories of 13.44 to 14.70 MJ/kg twice daily (06:00 and 14:00 h). All nursing sows were fed 6 to 7 kg of feed daily; the feed was composed of 18% concentrated crude protein and 17.52 MJ/kg, and was divided into four feeding times (06:00, 10:00, 14:00, and 18:00 h). The piglets were weaned between 22 and 28 days of age. Sow and litter records were collected between 2014 and 2019. The dataset consisted of sow identification, sow birth date, sow breed, sire identification, dam identification, parity, farrowing date, number of piglets born alive, number of piglets weaned, and litter weight at weaning. The database for the genetic evaluation of Danish pig genetics was fitted using PEST [9] and VCE6 software [10]. The data structure used in this study is presented in Table 1.

The best linear unbiased prediction (BLUP) procedure was applied for the genetic evaluation of purebred sows based on the reproduction traits number born alive (NBA), number weaned (NW), and litter weight on the 21st day (LW21), which were combined into the sow productivity index (SPI):

$$\text{SPI}=100+\frac{25}{\text{SD}}(3.09\times {\text{EBV}}_{\text{NBA}}+1.72\times {\text{EBV}}_{\text{NW}}+0.17\times {\text{EBV}}_{\text{LW2}1})$$

For young boars and gilts, the maternal line selection index (MLI) was applied in the evaluation:

$$\text{MLI}=100+\frac{25}{\text{SD}}(3.09\times {\text{EBV}}_{\text{NBA}}+1.72\times {\text{EBV}}_{\text{NW}}+0.17\times {\text{EBV}}_{\text{LW2}1}-0.27\times {\text{EBV}}_{\text{D}100}-0.17+{\text{EBV}}_{\text{BF100}})$$

where, EBV_NBA_, EBV_NW_, EBV_LW21_, EBV_D100_, and EBV_BF100_ are the estimated breeding values of NBA, NW, LW21, days of age to 100 kg (D100), and backfat thickness (ML100). SD is a breed-specific standard deviation. The average values of these indexes are 100 for each test group, with a SD of approximately 25.

### Statistical analysis

Variance components, heritability, and correlations for litter traits were estimated using the restricted maximum likelihood method with VCE6 software [10]. The breeding values of these traits were estimated using the BLUP procedure with PEST software [9] with the following repeatability model:

y=Xb+Za+Wp+e

Where y is a vector of observations for NBA, NW, and LW21, Vector **b** represents fixed effects, including HYS effect (herd×year×season interaction by birthdate), housing fixed effect (closed or opened), effect of age (months) of sow at farrowing treated as the covariant for litter traits with a regression coefficient. Vectors **a**, **p**, and **e** represent random animal genetic effects, random permanent non-genetic effects, and random residual effects, respectively. **X**, **Z**, and **W** are incidence matrices associating vectors **b**, **a**, and **p**, respectively, with vector y.

Variances were defined as follows:

$$\text{var}\;\left[\begin{array}{c} a\\ p\\ e \end{array}\right]=\left[\begin{array}{ccc} A\sigma_{a}^{2} & 0 & 0\\ 0 & I_{\mathrm{pe}}\sigma_{\mathrm{pe}}^{2} & 0\\ 0 & 0 & I_{e}\sigma_{e}^{2} \end{array}\right]$$

Where ***A*** is the additive relationship matrix; ***I****_pe_*, ***I****_e_* are identity matrices; and $\sigma_{a}^{2},\sigma_{\mathrm{pe}}^{2}$, and $\sigma_{e}^{2}$ are animal additive genetic, permanent environmental, and residual variances, respectively.

The genetic trends of the additive genetic values were determined by linear regression of the average predicted breeding values of NBA, NW, and LW21 traits, using INDEX as a function of dam’s birth year between 2013 and 2018.

## RESULTS AND DISCUSSION

### Phenotypic parameters

The three reproduction traits NBA, NW, and LW21 were analyzed in this study. Table 2 presents the number of sows and litter records, arithmetic means, SD, and minimum and maximum values for each litter trait. A total of 936 Yorkshire and 973 Landrace sows born between 2013 and 2018 were recorded with 3361 and 3161 litters, respectively. The average number of litters per sow was 3.25 litters in Yorkshire and 3.59 litters in Landrace. The average NBA and NW values were 12.7 and 12.1 in Yorkshire pigs and 12.9 and 12.0 in Landrace pigs, respectively. These results indicate a mean pre-weaning mortality of 4.9% in the Yorkshire herd and 7.5% in the Landrace herd. The LW21 values were 65.6 kg in Yorkshire pigs and 66.8 kg in Landrace pigs. Compared to the productivity for the top five pig farms in the DanBred system, the phenotype of litter traits in this study was reduced by 2.0 to 2.8 piglets per litter for NBA and 1.2 to 2.0 piglets for NW.

### Genetic variance and heritability

The genetic variance and heritability of litter traits are presented in Table 3. There were differences in the estimates of variance components between the two breeds for all three studied traits. Especially for NBA, the additive genetic variance was considerably larger in the Yorkshire herd (2.2688) than in the Landrace herd (1.3010). For the NW and LW21 traits, the differences were negligible for all components of genetic, residual, and phenotypic variances. In both breeds, the genetic variances in NBA and LW21 were sufficiently large, indicating an improvement through selection.

As these estimates depend on the population, study time, and data collected for each trait, it is difficult to interpret the differences in variance component estimates obtained in different studies. For the NBA in both breeds, the estimates of additive genetic variance in this study were considerably larger than those in some previous studies [4,11–13], but were only slightly larger than those in other studies [14,15]. For LW21, the estimates of additive genetic variance were approximately the same as those reported in previous studies [4,12]. Furthermore, these differences in genetic variances between the two breeds resulted in differences in the estimates of heritability for NBA, that is, 0.21 for Yorkshire pigs and 0.12 for Landrace pigs (Table 3). For the NW and LW21 traits, the differences in the estimates of heritability between Yorkshire and Landrace were insignificant (0.04 and 0.03 for NW; 0.12 and 0.13 for LW21).

Some differences in the heritability of litter traits were observed between this study and previous studies. According to a previous study, the heritability estimate was 0.22 for NBA, which is consistent with that obtained in the present study for Yorkshire pigs (0.21) [16]. In contrast, other studies estimated lower heritability for NBA (0.08) and LW21 (0.07) [17]. Similarly, certain studies have reported relatively small estimates of heritability, ranging from 0.10 to 0.08 for NBA, 0.04 to 0.05 for NW, and 0.08 to 0.09 for LW21 in Yorkshire and Landrace pigs, respectively [4,11].

For Polish pigs, a previous study reported heritability estimates of 0.023, 0.027, and 0.030 in the Polish Landrace line and 0.061, 0.058, and 0.075 in the PL-23 line for NBA, NW21, and LW21, respectively [18]. For Irish Yorkshire and Landrace pigs, a study, based on data collected from 1992 to 2010, estimated a very small heritability value of 0.023 for the NBA [14]. In China, another study indicated heritability of 0.12 in Yorkshire pigs and 0.11 in Landrace pigs for the NBA [15].

For Landrace and Large White pigs raised in a tropical climate in Thailand, a very low heritability of 0.05 was reported for both NBA and litter weight weaned (LWW) [12]. Recently, very small estimates of heritability were reported for Bisaro pigs in Portugal, that is, 0.015 for NBA and 0.010 for NW [13]. Based on a Gaussian model and a small and closed population of Landrace pigs from a research center, smaller heritability estimates of 0.03 and 0.01 were reported for NBA and NW, respectively, which may reduce the genetic variation [19].

For different litters of Dutch Landrace pigs, heritability estimates of 0.084 and 0.089 for NBA in litter one and litters two to six, respectively, have been reported [20]. Conversely, heritability estimates for purebred German Landrace pigs were 0.05 and 0.027 for NBA in litter one and litters two to ten, respectively [21]. In addition, for crossbred German Landrace×German Large White pigs, a heritability estimate of 0.10 has been reported for NBA based on the data of 2,602 litters from 1,102 different sows born between 2007 and 2015 [22].

Even in studies related to the same genetic groups as Danish pigs, research conducted in different periods of time showed different results. For instance, data collected by a study on Danish Landrace and Yorkshire pigs in Denmark (from 1985 to 1989) reported heritability estimates of 0.11 to 0.14 in Landrace pigs and 0.10 to 0.11 in Yorkshire pigs for the total number of piglets born [23]. Moreover, a previous study on the genetic variance and covariance components for litter size and litter weight in Danish Landrace pigs, using a multivariate mixed model, revealed heritability estimates of 0.03, 0.05, and 0.07 for NBA, NW, and LWW, respectively [24]. During 2002 to 2004, with data collected from 43 nuclear farms of Danish Yorkshire and Landrace pigs, Su et al [25] reported heritability estimates of 0.078 for NBA and 0.09 for the number weaned at 21 days of age (N3W) in Landrace pigs and 0.05 for NBA and 0.065 for N3W in Yorkshire pigs. For the same animal population used in this study, heritability estimates of 0.07, 0.03, and 0.10 for NBA, NW, and LW21, respectively, in Landrace pigs, and 0.13, 0.09, and 0.14, respectively, for the corresponding traits in Yorkshire pigs have been reported [8].

Thus, the heritability estimates for litter traits depend on the genetic structure of the studied populations, study period, dataset size, and estimation methods. In this study, the heritability of NBA was low (0.12 to 0.21), but still significantly higher than that reported for the same genetic groups of Danish pigs. This means that improvement in the studied traits in Danish Landrace and Yorkshire pigs will be feasible in the coming years through selection.

### Genetic correlations

Genetic, environmental, and phenotypic correlations between the NBA and NW (NBA-NW), NBA and LW21 (NBA-LW21), and NW and LW21 (NW-LW21) are indicated in Table 4. In general, all correlations among the studied traits were positive in both Yorkshire and Landrace breeds; however, differences were observed in the correlation levels and absolute values for each pair of traits. Between the NBA and NW, the genetic correlations were relatively strong in both breeds, at 0.77 and 0.60 for Yorkshire and Landrace, respectively, whereas both environmental and phenotypic correlations were moderate (0.32 to 0.37) for Yorkshire and Landrace breeds. Similarly, between the NW and LW21, the genetic, environmental, and phenotype correlations were quite strong in Yorkshire pigs (0.48 to 0.62) but stronger in Landrace pigs (0.6 to 0.71). Conversely, between the NBA and LW21, the genetic, environmental, and phenotype correlations were all low: 0.14 to 0.20 in Yorkshire breeds and 0.01 to 0.16 in Landrace breeds.

In previous studies, a large difference was observed in the genetic correlations between the NBA and NW, NBA and LW21, and NW and LW21 in pigs. These correlations depend on the studied traits, genetics, populations, time, and statistical methods. One of the main reasons for the differences between current study and others is the statistical model for litter traits with the additive genetic effect adjusted by sow’s age (months). Some studies have reported negative correlations between −0.07 and −0.99 in Australian pigs and Polish Landrace pigs [17,18]. Another study reported positive correlations for these pairs of traits, with values ranging from 0.14 to 0.75 in Yorkshire pigs and 0.14 to 0.73 in Landrace pigs [4]. A study in Thailand indicated that the genetic correlation between the NBA and LWW was very weak (~0.08) in Large White and Landrace pigs [12]. Conversely, some researchers have reported a strong positive genetic correlation between the NBA and NW, at 0.91 to 0.96 in Large White and Landrace pigs in Czechoslovakia and 0.95 in Bisaro pigs in Brazil [2,13].

For pig genetics originating from Denmark, the genetic variance and covariance components for litter size and litter weight in Danish Landrace pigs have been estimated using a multivariate mixed model, which indicated genetic correlations of 0.79 between the NBA and NW as well as between the NBA and LW21 [24]. Furthermore, data collected from 43 nuclear farms of Danish Yorkshire and Landrace pigs during 2002 to 2004 indicated genetic correlations between the NBA and N3W of 0.87 in Yorkshire pigs and 0.72 in Landrace pigs [25]. For the same animal population used in this study, strong positive genetic correlations (0.37 to 0.60) for NBA-NW and NW-LW21 have been reported for Landrace pigs and very strong positive genetic correlations (0.84 to 0.90) have been reported for NBA-NW, NBA-LW21, and NW-LW21 traits in Yorkshire pigs [8].

As discussed above, the genetic correlations estimated in this study between NBA and NW and between NBA and LW21 were positive and relatively high in both breeds, which is consistent with the results of previous studies, especially those conducted in the same Danish pig genetics. Thus, there is an advantage in improving NBA and NW simultaneously in Danish Landrace and Yorkshire pigs through genetic evaluation programs.

### Genetic trends

**Figure 1:**
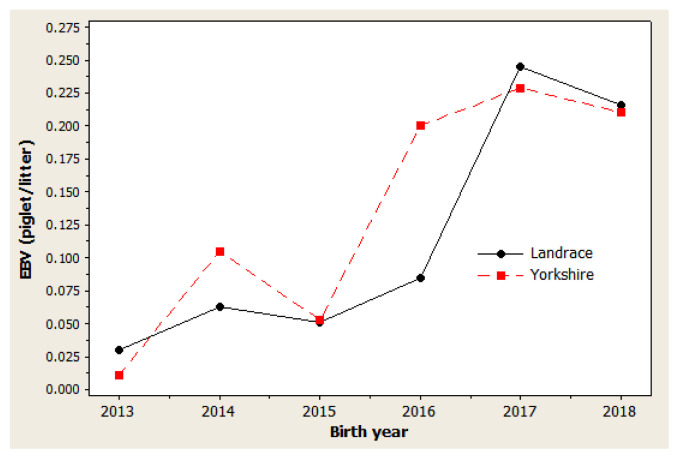
Genetic trend of number born alive in Danish Landrace and Yorkshire pigs by year of birth. Regression coefficients were 0.0431 piglet/yr (p = 0.021) for Landrace and 0.0434 piglet/yr (p = 0.019) for Yorkshire pigs.

**Figure 2:**
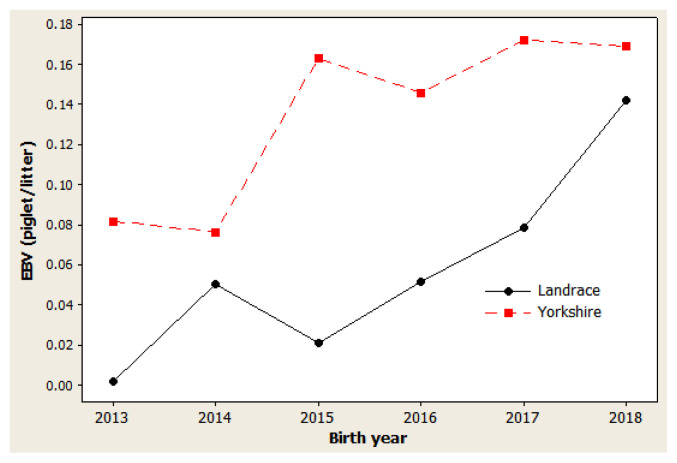
Genetic trend of number weaned in Danish Landrace and Yorkshire pigs by year of birth. Regression coefficients were 0.0233 piglet/yr (p = 0.019) for Landrace and 0.0202 piglet/yr (p = 0.029) for Yorkshire pigs.

**Figure 3:**
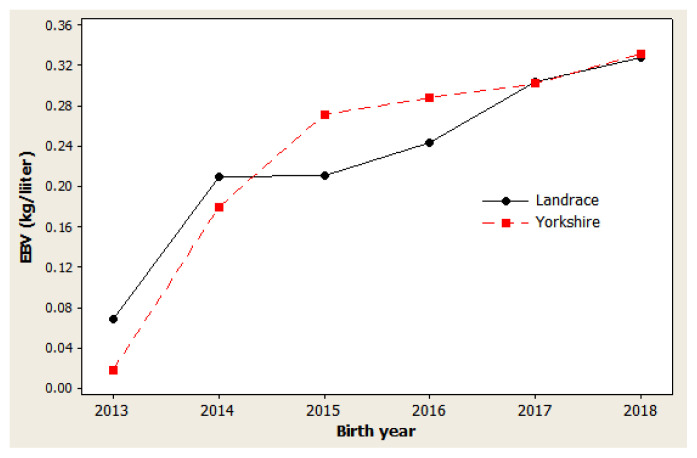
Genetic trend of litter weight at the 21st day in Danish Landrace and Yorkshire pigs by year of birth. Regression coefficients were 0.0461 kg/yr (p = 0.005) for Landrace and 0.0557 kg/yr (p = 0.016) for Yorkshire pigs.

The genetic trend estimates for selected traits reveal whether selection decisions made during the breeding program resulted in effective improvements. This is important for assessing and adjusting current breeding schemes. For NBA (Figure 1), negative trends were observed in 2014 to 2015 and in 2017 to 2018 for both Landrace and Yorkshire herds. However, in 2013 and 2018, the genetic trend was positive for this trait, with large coefficients of determination (R^2^) values: 77.5% in Landrace and 78.5% in Yorkshire. For NW (Figure 2), negative trends were observed for the Landrace herd in 2014 to 2015 and for the Yorkshire herd in 2013 to 2014. Similarly, the genetic trend for NW was positive during 2013 and 2018, with large R^2^ values of 78.6% and 73.4% in the Landrace and Yorkshire herds, respectively. For LW21 (Figure 3), a continuously positive genetic trend was observed during 2013 and 2018, with a large R^2^ value of 88.4% in Landrace and 80.0% in Yorkshire. The annual regression coefficients for NBA (Figure 1), NW (Figure 2), and LW21 traits (Figure 3) were 0.0431, 0.0233, and 0.0461 in Landrace and 0.0434, 0.0202, and 0.0557 in Yorkshire, respectively, which indicated a relatively small annual genetic gain for these traits during 2013 and 2018.

**Figure 4:**
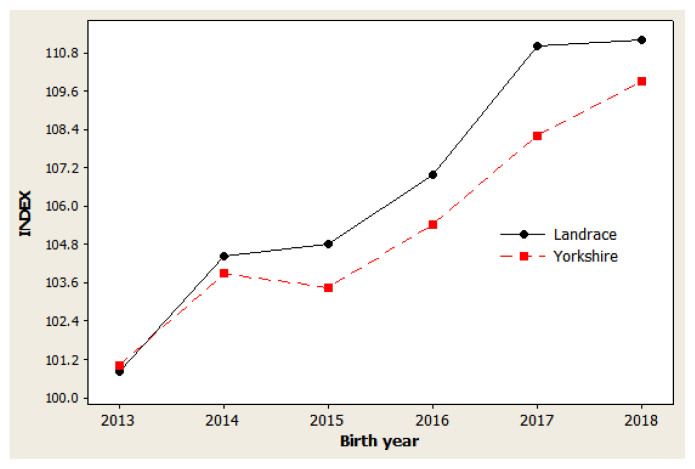
Genetic trend of sow productivity index in Danish Landrace and Yorkshire pigs by year of birth. Regression coefficients were 2.11 index point/yr (p = 0.001) for Landrace and 1.70 index point/yr (p = 0.001) for Yorkshire pigs.

As presented in Figures 1, 2, and 3, different directions of mean EBV changes were observed at different periods of time for each trait; however, for the selection INDEX combining all three traits, genetic trends were predominantly positive in both Yorkshire and Landrace pigs (Figure 4). This was indicated by the steadily linear regressions with R^2^ values of close to 100%: 94.9% for Landrace and 94.3% for Yorkshire. The average annual INDEX values increased by 2.1092 in Landrace pigs and 1.6976 in Yorkshire pigs, indicating the genetic progress of INDEX in the studied animals.

Regarding reproduction traits in pigs, different programs of genetic improvement may result in different levels of genetic progress, which can depend on the selection strategy, population size, evaluation methods, and procedures. In French Large White pigs, the annual genetic trends from 1977 to 1998 revealed estimates of 0.15 and 0.13 piglets for total piglets born and NBA, respectively [26]. In Polish Landrace pigs, negative NBA changes were observed in 1975 to 1983 and 1985 to 1992 in PL pigs from 1973 to 1999 [18]. Conversely, positive changes were observed for both NBA and LWW in PL-23 pigs during 1983 and 1999. The estimated values of total genetic progress were 0.17 piglets for NBA, 0.10 piglets for NW21, and 0.54 kg for LW21 [18].

In the United States, the estimated average genetic trends of 0.029, 0.008, and 0.279 have been reported for NBA, NW, and LW21, respectively, in Yorkshire pigs [4]. These values were lower in Landrace pigs, averaging 0.021 for NBA, 0.0081 for NW, and 0.004 for LW21. In another study, the regression coefficients of mean EBVs between the 12th and 27th generations of a Large White and Landrace composite population from 1981 to 2009 have been reported to be 0.025 and 0.016 piglets per generation for NBA and NW in the selection lines, respectively [27]. In Brazil, a study indicated a low degree of genetic progress for NBA in Large White pigs (0.028 piglets/L/yr), which was attributed to the low heritability of reproductive traits [28]. For Danish Landrace and Yorkshire populations in current study, the selection program had applied for the indices SPI and MLI constructed similarly to the pig breeding programs in United States and Brazil. But the economic weight of NBA was much higher than that of NW and LW21 traits including these selection indices. This means that the genetic selection for two these pig populations had focused mainly on NBA trait during past five years. It may be the reason for significantly higher genetic progress for litter traits in current study as compared with papers discussed above, especially for NBA in both Landrace and Yorkshire breeds.

However, the genetic progress for litter traits in this study was much lower as compared with some pig populations selected in China or Thailand. For instance, a study on 6,363 sows in Chinese Landrace pigs during 2000 to 2012 reported an annual genetic gain of 0.08 piglets per litter per year for NBA [29]. Another study of total 3,109 Landrace and Yorkshire sows in Northern Thailand indicated that genetic trends were positive and significant for all traits during 1989 and 2013, that is, 0.16 for NBA, 0.12 for NW, and 1.23 for LWW [30]. These differences could be due the selection also being aimed at growth rate and backfat thickness, beside litter traits and being applied to smaller animal populations (1,909 sows in total) as well as the within-herd selection scheme in current study.

As mentioned in method section, a breeding program has set up for Danish Landrace and Yorkshire pigs in this study since 2013 with objectives to improve litter traits and to maintain growth rate as well as backfat thickness. The BLUP statistical procedure was applied for estimating the breeding values and genetic evaluation for selective traits. The MLI was constructed including growth rate, backfat thickness and three litter traits to select young boars and gilts after finishing individual performance test. And then, SPI including three litter traits was applied for sow’s productivity evaluation. During 2013 and 2018, the annual genetic progress determined for litter traits NBA, NW, and LW21 in two these populations was relatively small. However, the positive values of these genetic trends confirmed the validity of the breeding program and the suitability of the selection strategy for these genetics. The large number of selected traits in the breeding program, the small population size, and the within-herd selection scheme could be the main factors influencing the low selection efficiency and slow genetic progress in these genetics. Therefore, the BLUP procedure should be retained for further genetic evaluation, and the selective population size should be increased by a cross-herd selection, which will enable more precise estimates of the breeding values.

## IMPLICATIONS

Low heritability values were observed for litter traits in Danish Landrace and Yorkshire pigs. The genetic variances in number born alive and litter weight at the 21st day were sufficiently large for improvements these traits through selection. The favorable genetic correlations showed that the simultaneous improvements in the litter traits number born alive, litter weight at the 21st day, number weaned, and litter weight at the 21st day could be promising breeding goals for these pig genetics. The positive genetic trends estimated for the additive genetic values of the selected traits indicated that the current breeding system has achieved favorable results. From these findings, it is evident that the best linear unbiased prediction procedure should be implemented in both within-farm and across-farm genetic evaluation programs.

## Figures and Tables

**Table 1:** Structure of data collected for the genetic analysis of Danish Yorkshire and Landrace pigs

Birth year	Yorkshire			Landrace		
	
	Number of boars	Number of sows	Number of litters	Number of boars	Number of sows	Number of litters
2013	40	102	478	39	113	538
2014	16	113	526	27	112	573
2015	30	251	1,064	26	155	645
2016	15	284	977	23	249	861
2017	7	156	286	1	267	467
2018	3	30	30	4	77	77
Total	111	936	3,361	136	973	3,161

**Table 2:** Phenotypic parameters of litter traits in Danish Yorkshire and Landrace pigs between 2014 and 2019

Items	Yorkshire	Landrace
Number of sows	936	973
Number of litters	3,361	3,161
Number of litters per sow (L)	3.25	3.59
Number born alive (piglets, mean±SD) (minimum to maximum)	12.7±3.5 (2 to 23)	12.9±3.5 (2 to 24)
Number weaned (piglets, mean±SD) (minimum to maximum)	12.1±2.9 (2 to 21)	12.0±3.2 (2 to 19)
Litter weight at 21st day (kg, mean±SD) (minimum to maximum)	65.6±11.1 (33.1 to 114)	66.8±11.1 (36.9 to 120)

**Table 3:** Variance components and heritability of number born alive (NBA), number weaned (NW), and litter weight at the 21st day (LW21) in Danish Yorkshire and Landrace pigs

Genetics/variance components and heritability	Litter traits		

	NBA	NW	LW21
Yorkshire pigs			
Additive genetic effects (σ^2^_a_)	2.2688	0.2562	14.2609
Permanent environmental effects (σ^2^_pe_)	0.0325	0.1939	6.0780
Residual effects (σ^2^_e_)	8.4360	5.4530	104.1830
Phenotypic variance (σ^2^_P_)	10.7370	5.9030	124.5220
Heritability (h^2^±SE)	0.21±0.02	0.04±0.02	0.11±0.02
Landrace pigs			
Additive genetic effects (σ^2^_a_)	1.3010	0.2230	15.6680
Permanent environmental effects (σ^2^_pe_)	0.1514	0,0231	3.3713
Residual effects (σ^2^_e_)	9.8740	6.4230	100.0920
Phenotypic effects (σ^2^_P_)	11.3270	6.5890	119.131
Heritability (h^2^±SE)	0.12±0.02	0.03±0.02	0.13±0.02

NBA, number born alive; NW, number weaned; LW21, litter weight at the 21st day; SE, standard error.

**Table 4:** Genetic, environmental, and phenotypic correlations among the litter traits of number born alive, number weaned, and litter weight at the 21st day in Danish Yorkshire and Landrace pigs

Genetics/pair of litter traits	Genetic correlation (rA±SE)	Environmental correlation (rE±SE)	Phenotypic correlation (rP±SE)
Yorkshire pigs			
NBA - NW^1)^	0.77±0.12	0.34±0.02	0.37
NBA - LW21^2)^	0.20±0.10	0.14±0.02	0.15
NW - LW21^3)^	0.48±0.13	0.62±0.01	0.62
Landrace pigs			
NBA - NW^1)^	0.60±0.16	0.32±0.02	0.34
NBA - LW21^2)^	0.01±0.13	0.16±0.02	0.13
NW - LW21^3)^	0.71±0.12	0.67±0.01	0.65

SE, standard error; NBA, number born alive; NW, number weaned; LW21, litter weight at the 21st day.

1) NBA – NW, between NBA and NW.

2) NBA - LW21, between NBA and LW21.

3) NW - LW21, between NW and LW21.
